# Fine-needle aspiration of follicular lesions of the thyroid. Diagnosis and follow-Up

**DOI:** 10.1186/1742-6413-3-9

**Published:** 2006-04-07

**Authors:** M Salih Deveci, Güzin Deveci, Virginia A LiVolsi, Zubair W Baloch

**Affiliations:** 1 Department of Pathology and Laboratory Medicine, University of Pennsylvania Medical Center, Philadelphia, PA, USA

## Abstract

The differential diagnosis of a follicular lesion/neoplasm in thyroid FNA specimens includes hyperplastic/adenomatoid nodule, follicular adenoma and carcinoma, and follicular variant of papillary thyroid carcinoma. In our laboratory we separate follicular lesions of thyroid into hyperplastic/adenomatoid nodule (HN), follicular neoplasm (FON) and follicular derived neoplasm with focal nuclear features suspicious for papillary thyroid carcinoma (FDN).

This study reports our experience with 339 cases diagnosed as FON and 120 as FDN.

All cases were evaluated for histologic diagnosis, age, sex and size of the nodule. Histopathologic follow-up was available in all cases. The malignancy rate was 22% (74/359) and 72% (86/120) for cases diagnosed as FON and FDN, respectively. In the FON category almost half of the malignant cases were papillary carcinoma. The risk of malignancy was higher in patients younger than 40 yr (53% vs. 30%) than in patients 40 year or more years old and greater in males (41% vs. 33%) than females. No statistically significant relationship was noted between the sizes of the nodules and benign vs. malignant diagnosis.

According to this study it is important to divide follicular patterned lesions of thyroid into FON and FDN in the cytology specimens due to significantly different risk of malignancy (22% vs. 72%). In addition, clinical features, including gender and age can be part of the decision analysis in selecting patients for surgery.

## Introduction

Fine-needle aspiration (FNA) has been widely accepted as an initial step in the management of thyroid nodules. It is relied upon to distinguish benign from neoplastic/malignant thyroid nodules, thus, influencing therapeutic decisions [1,2]. However, the diagnostic efficacy of FNA declines sharply in the diagnosis of follicular patterned lesions of thyroid, i.e. separating hyperplastic/adenomatoid nodule, follicular adenoma (FA), follicular carcinoma (FCA) and follicular variant of papillary carcinoma (FVPTC) [3-5]. Most of these cases are diagnosed as follicular lesion/neoplasm and surgical excision is recommended for definite diagnosis on histopathologic examination [5,6].

It has been shown that the malignancy rate in cases diagnosed as follicular lesion/neoplasm (FON) is approximately 20% [6-9]. This high rate of benign lesions undergoing surgery is because FNA cannot distinguish between follicular adenoma and carcinoma on the basis of cyto-morphology [10]. This distinction is made by demonstrating capsular and/or vascular invasion on histopathologic examination [10]. Similarly the cytologic diagnosis of follicular variant of papillary carcinoma can be challenging due to overlapping cytologic features, with both benign and malignant follicular patterned lesions of the thyroid [11,12]. We have shown in previously published studies that these cases can be distinguished from those diagnosed as follicular neoplasm on the basis of subtle nuclear changes suggestive of papillary thyroid carcinoma [5,11]. We classify such lesions as follicular derived neoplasm with features suspicious for papillary carcinoma. The malignancy rate in such lesions is 70–75% i.e. much higher than seen in cases diagnosed as follicular neoplasm [5].

In this study, we report on our experience with 459 lesions diagnosed as "follicular neoplasm (FON)" and "follicular derived neoplasm with features suspicious for papillary carcinoma (FDN)"; all lesions had histopathologic follow-up.

## Materials and methods

At University of Pennsylvania Medical Center (UPMC) 5800 cases underwent ultrasound guided thyroid FNA from October 1999–May 2005. Three hundred and thirty-nine cases diagnosed as follicular neoplasm and 120 as follicular derived neoplasm suspicious for follicular variant of papillary thyroid carcinoma with histopathologic follow-up were selected for this study.

All thyroid aspirations were performed under ultrasound guidance by an endocrinologist and a radiologist. The FNA was performed using a 25-gauge needle attached to a 10-ml syringe. On average, 2 passes were made in each nodule, resulting in two air-dried and two alchohol-fixed smears. Diff-Quik (Harleco, Gibbstown, NJ) stained air-dried smears were used for on-site evaluation and alcohol-fixed smears were stained by modified Papanicolaou technique. The needle was rinsed in Normosol^® ^(Abbott Laboratories, Chicago, IL) for cellblock and Milipore filter (Millipore, Bedford, MA) preparation.

The adequate FNA specimen was defined as containing at least 4–6 cell groups on 2 slides, with 10–20 follicular cells in each group. The cases were diagnosed as FON and FDN according to previously published criteria.^5–6 ^In short, the cases diagnosed as FON showed a monotonous population of follicular cells arranged in cohesive groups with nuclear overlapping and crowding in a background of scant colloid. The cases diagnosed as FDN showed follicular cells arranged in loosely cohesive monolayer sheets and follicular groups, focally the cells demonstrated nuclear elongation, chromatin clearing and intranuclear grooves in a background of watery and thick colloid. All patients underwent either lobectomy, or total thyroidectomy with an intraoperative consultation in cases diagnosed as FDN on cytology. All demographic data was obtained from UPMC laboratory information system.

## Results

The clinicopathologic and histopathologic features are illustrated in Tables 1, 2, 3.

**Table 1:** Malignancy Rates in Cases Diagnosed as FON and FDN

***FNA Diagnosis***		***Histopathologic Correlation***	
		
		**BENIGN**	**MALIGNANT**
**FON**	(n = 339)	265 (78%)	74 (22%)
**FDN**	(n = 120)	34 (28%)	86 (72%)

Total	(n = 459)	299 (65%)	160 (35%)

FON – Follicular neoplasm, FDN-Follicular-derived neoplasm.

**Table 2:** *Histopathologic Correlation in Cases of FON and FDN*.

***FNA Diagnosis***		***Histologic Diagnosis***					
		
		**NG**	**FA**	**FVPTC**	**PTC**	**FCA**	**HCCA**
**FON**	(n=339)	120 (35%)	145 (43%)	36 (11%)	8 (2%)	21 (6%)	9 (3%)
**FDN**	(n = 120)	24 (20%)	10 (8%)	65 (54%)	14 (12%)	5 (4%)	2 (2%)

Total	(n = 459)	144 (31%)	155 (33%)	101 (22%)	22 (5%)	26 (6%)	11 (2.5%)

FDN-follicular-derived neoplasm suspicious for papillary carcinoma, FON-follicular neoplasm, NG-nodular goiter, FA-follicular adenoma, PTC-classic papillary carcinoma, FVPTC-follicular variant papillary carcinoma, FCA-follicular carcinoma, HCCA-Hürthle cell carcinoma.

**Table 3:** *Clinico-pathologic Features of Cases Diagnosed as FON and FDN on FNA*.

FNA Diagnosis	Histologic Diagnosis					
	
	***NG***	***FA***	***FVPTC***	***PTC***	***FCA***	***HCCA***
***SEX***						
****Female (n = 372)	120	128	82	16	18	8
Male (n = 87)	24	27	19	6	8	3
***AGE*** (ave.: 52 year)	*55 yr*	*53 yr*	*48 yr*	*48 yr*	*49 yr*	*44 yr*
< 45 Yr (n = 126)	25	38	42	10	7	4
46-60 Yr (n =207)	75	69	38	6	14	5
> 60 Yr (n = 126)	44	48	21	6	5	2
***SIZE*** (ave.: 2.3 cm)	*2.2 cm*	*2.2 cm*	*2.4 cm*	*1.7 cm*	*2.4 cm*	*3.4 cm*
< 1.5 cm (n = 138)	48	48	24	9	7	2
1.5–3 cm (n = 232)	73	78	52	11	13	5
> 3 cm (n = 89)	23	29	25	2	6	4

**Total **(n = 459)	144 (31%)	155 (33%)	101 (22%)	22 (5%)	26 (6%)	11 (2.5%)

FDN-follicular-derived neoplasm suspicious for papillary carcinoma, FON-follicular neoplasm, NG-nodular goiter, FA-follicular adenoma, PTC-classic papillary carcinoma, FVPTC-follicular variant PTC, FCA-follicular carcinoma, HCCA-Hürthle cell carcinoma

The patients ranged in age from 16–87 yr (average age, 52 yr); there were 372 females and 87 males. The size of the aspirated lesions was determined by the ultrasound and ranged from 0.6–8.5 cm (average size 2.3 cm, (only one cystic nodule measured 8.5 cm); 138 nodules were less than 1.5 cm (only 6 nodules measured less than 1 cm); 232 nodules ranged in size from 1.5–3 cm and, 89 were greater than 3 cm in size.

Surgical follow-up was available in all cases; 299 cases (299/459 65%) were classified as benign and 160 (160/459 35%) as malignant on histologic examination.

Of the 339 cases diagnosed as FON on cytology, histologic follow-up was benign in 265 (78%) and malignant in 74 (22%) cases. The malignant diagnoses included follicular variant of papillary carcinoma 36, classic papillary carcinoma 8, follicular carcinoma 21 and Hurthle cell carcinoma 9 cases.

Of the 120 cases diagnosed as FDN, the histologic follow-up was benign in 34(28%) and malignant in 86 (72%) cases. The malignant diagnoses included FVPTC 65, PTC 14, FCA 5 and Hurthle cell carcinoma 2 cases. Intraoperative consultation was requested in all cases of FDN; 24 cases (30%) were diagnosed as papillary carcinoma on frozen section and touch preparation.

The average size of the benign nodules was 2.2 cm, whereas, the average size of classic papillary carcinoma was 1.7 cm, FVPTC and FCA 2.4 cm and Hurthle cell carcinoma 3.4 cm. The rate of malignancy in females was 33% as compared to 41% in males.

## Discussion

To date several published series have documented the difficulty in diagnosing follicular patterned lesions of the thyroid in cytology preparations [5,13-15]. The histologic follow-up of cases diagnosed as follicular lesions of neoplasm includes hyperplastic/adenomatoid nodules, follicular adenoma, follicular carcinoma and follicular variant of papillary carcinoma [6,15]. An earlier study published by Schlinkert et al from Mayo clinic showed that only 12% cases diagnosed as "suspicious for follicular neoplasm" on FNA were malignant on histologic follow-up. Interestingly, 27% were papillary carcinomas (majority were follicular variant) [4]. Tuttle et al reported malignancy rate of 21% in their series of 149 patients diagnosed as follicular neoplasm on cytology [8]. In our previous study the malignancy rate was 31% in 122 patients diagnosed as follicular neoplasm and almost half of these cases were follicular variant of papillary carcinoma.

In view of these above-mentioned studies we retrospectively reviewed the cases of FVPTC and found that some cases are under-diagnosed as follicular neoplasm due to the paucity of nuclear features of papillary carcinoma, an exact reflection of what is seen in surgical pathology of some cases of FVPTC [3,16]. Similar findings have been reported by other authors [17-19]. In view of these studies at our institution the thyroid FNA specimens are classified as benign, neoplastic, FDN/suspicious for papillary carcinoma, definitely malignant and non-diagnostic.

In the present study, 339 cases were diagnosed as follicular neoplasm; the malignancy rate in this group was 22% and half of the cases were FVPTC. These findings are similar to previously published studies. Of the 120 cases diagnosed as FDN 72% (86 cases) were malignant and papillary carcinoma was present in 92% (79/86) of cases.

**Figure 1:**
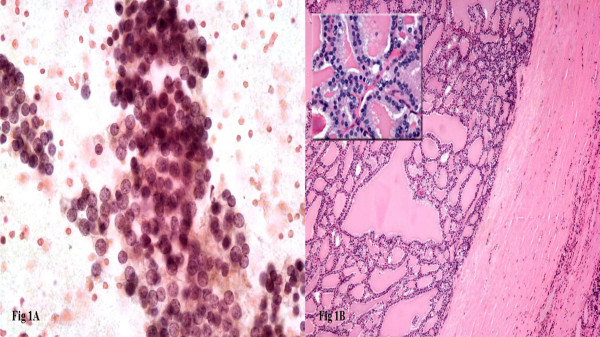
Monotonous population of follicular cells arranged in cohesive follicular groups with nuclear overlapping and crowding, case diagnosed as follicular neoplasm (1A). Histologic follow-up showing follicular adenoma (thickly encapsulated follicular patterned lesion with no capsular or vascular invasion), the inset shows the same nuclear features as seen in cytology (1B).

It has been shown that thyroid nodules can be divided into high and low risk of malignancy on the basis of clinical characteristics. Schlinkert et al reported that findings of larger diameter, fixation of mass and younger age of patients were associated with high risk of malignancy [4]. According to study by Tyler et al patients greater than 50 years of age with a diagnosis of follicular neoplasm had a higher risk of malignancy as compared to patients younger than 50 years [9]. In a previous study of 122 patients diagnosed as FON we showed that patients aged 40 years or more had higher risk of malignancy than those younger than 40 years [6]. Interestingly, the current study which consists of a much larger group of patients and combines cases diagnosed as FON and FDN the malignancy rate was higher in patients younger than 40 years than those aged 40 years or higher (53% vs. 30%). No significant difference in malignancy rates was noted between thyroid nodules measuring less than 3 cm or equal to and greater than 3 cm. (Fig 1). These differences from the previous studies could be due to a higher number of cases diagnosed as papillary carcinomas (>70%) in the present study. Since, papillary carcinoma is common at younger age and can occur in any size as compared to follicular or Hurthle cell carcinoma.

The other significant predictor of malignancy in our study was sex of patient; malignant tumors were more common in males as compared to female patients (41% vs. 33%). In addition, though not statistically significant FCA was more common in males as compared to females (30% vs. 21%).

Frozen section is usually not recommended for the diagnosis of thyroid lesions [20]. Studies have shown that intraoperative consultation is of no value in the diagnosis of follicular carcinoma since its diagnosis is dependent upon invasion of tumor capsule and/or capsular vessels, which can be missed by limited sampling of tumor capsule on frozen section [20,21]. However, frozen section combined with intraoperative cytology has been shown to be of value in cases diagnosed as suspicious of papillary carcinoma in preventing two-step surgical excision (lobectomy followed by total thyroidectomy)[21]. At our institution, we recommend intraoperative consultation in cases diagnosed as FDN on cytology[5]. In the present study 36(30%) cases were diagnosed as papillary carcinoma on frozen section and intraoperative cytology.

It has been shown that molecular markers can be of value in the cytologic diagnosis of malignant lesions of thyroid. Ret-oncogene rearrangements are believed to be specific to papillary carcinoma of the thyroid, and this marker can be of value in identifying cases of FVPTC in thyroid FNA [22]. Recently, BRAF rearrangements have been reported in papillary carcinoma and shown to be specific to this tumor. Similar studies regarding detection of BRAF rearrangements in thyroid FNA specimens have shown promise as a diagnostic aid to morphology [22,23]. However, BRAF expression is less common in FVPTC as compared to classic papillary carcinoma; therefore, its use in the diagnosis of FVPTC in FNA specimens may be of limited value.

***In conclusion***, until a specific markers or panel of markers is devised which can effectively distinguishes between benign and malignant follicular lesions of the thyroid in FNA specimens morphology remains the gold standard. The category of follicular lesion/neoplasm can further modified by dividing these cases into two: lesions with and without subtle nuclear features of papillary carcinoma because of marked difference in malignancy rates (22% vs. 72%).

## Competing interests

The author(s) declare that they have no competing interests.

## Authors' contributions

ZWB conceived and designed the study, supervised the research team and data analysis by MSD and GD, and wrote the report with MSD, GD and VAL. All authors were involved in the critical revision of the manuscript drafts and approved the final version for publication.

**Figure 2:**
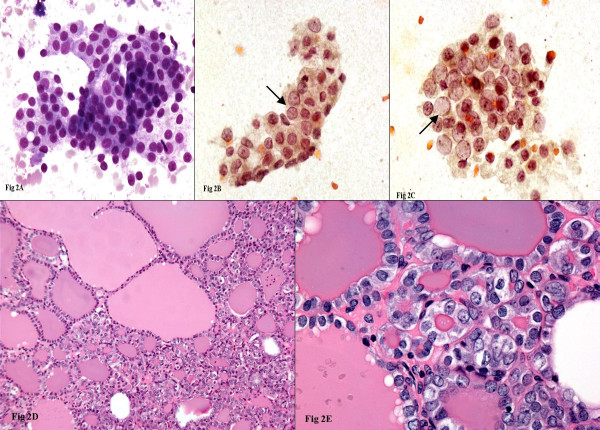
Diff-Quik^® ^stained smear demonstrating enlarged follicular cells in loosely cohesive groups (2A). Papanicolaou stained slides showing focal nuclear chromatin clearing and intranuclear grooves (arrows-Figs 2B-2C). Histologic follow-up showing follicular variant of papillary thyroid carcinoma, notice the nuclear features on high power (Figs 2D-2E).

**Figure 3:**
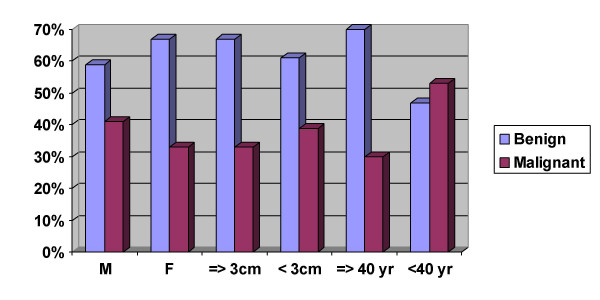
Correlation between benign and malignant diagnoses, size of the thyroid nodule, age and sex of the patient.
